# Supplementary figures and images for: Influenza A virus coinfection dynamics are shaped by distinct virus-virus interactions within and between cells
Source: PLoS Pathog. 2023 Mar 2;19(3):e1010978. doi: 10.1371/journal.ppat.1010978 (PMC10013887; doi:10.1371/journal.ppat.1010978)

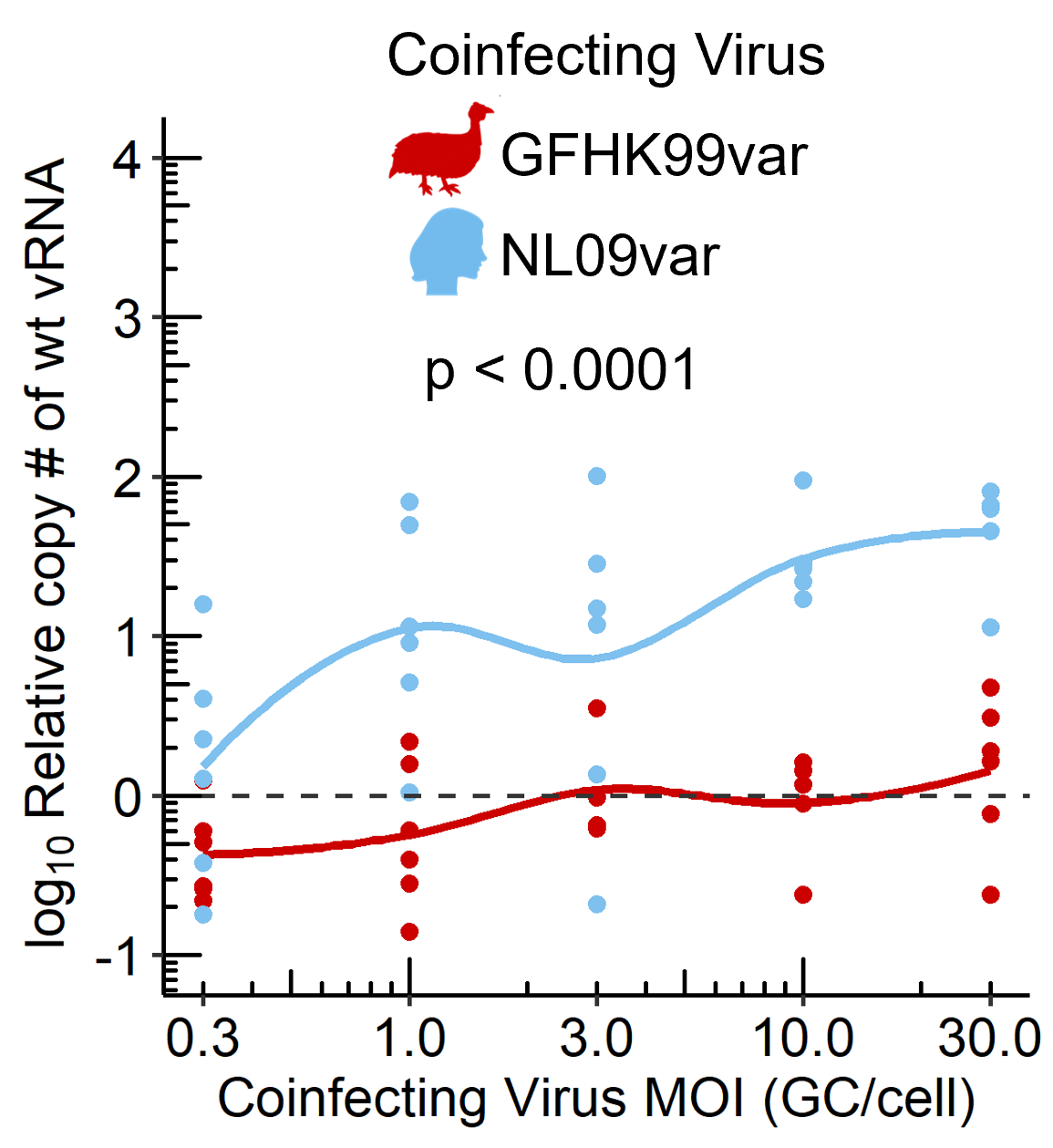

Supplement: S1 Fig — (Related to Fig 2) NHBE cells were infected with GFHK99wt virus at an MOI of 0.5 genome copies (GC)/cell and increasing doses of the homologous GFHK99var virus or the heterologous NL09 virus. The fold change in GFHK99wt vRNA copy number, relative to GFHK99wt-only control (dashed line), is plotted. Results of six biological replicates derived from two independent experiments are plotted and solid lines connect the means. Significance of differences between results obtained with the differing coinfecting viruses were evaluated by two-way ANOVA. (TIF) [file ppat.1010978.s001.tif]

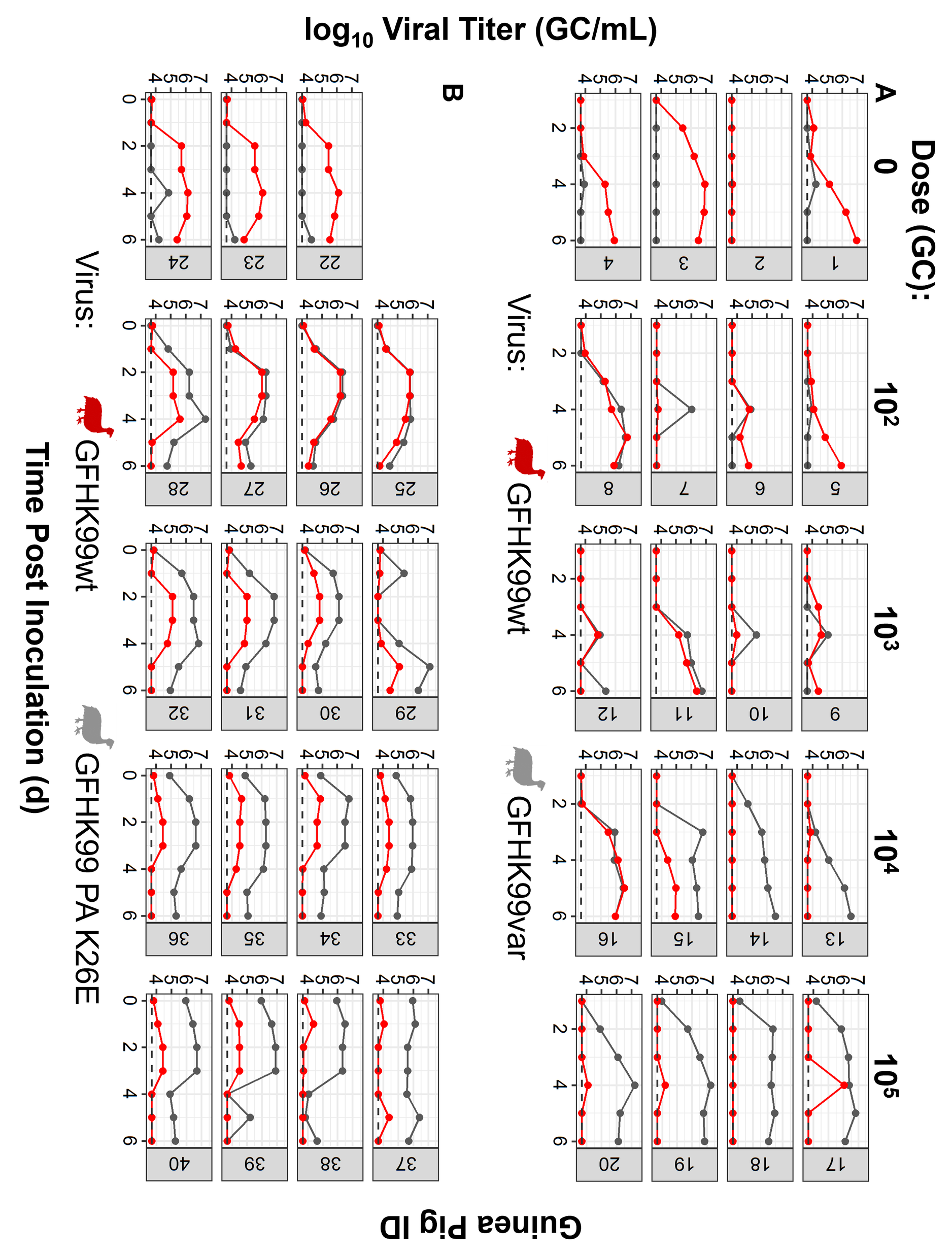

Supplement: S2 Fig — (Related to Fig 5A). Guinea pigs were infected with GFHK99wt at a dose of 103 GC and increasing doses of GFHK99var (A) or GFHK99 PA K26E (B) virus. The viral titer in nasal washes collected from each guinea pig is plotted and the limit of is indicated by the dashed line. Guinea pig ID numbers are shown in grey boxes appended to each facet. (TIF) [file ppat.1010978.s002.tif]

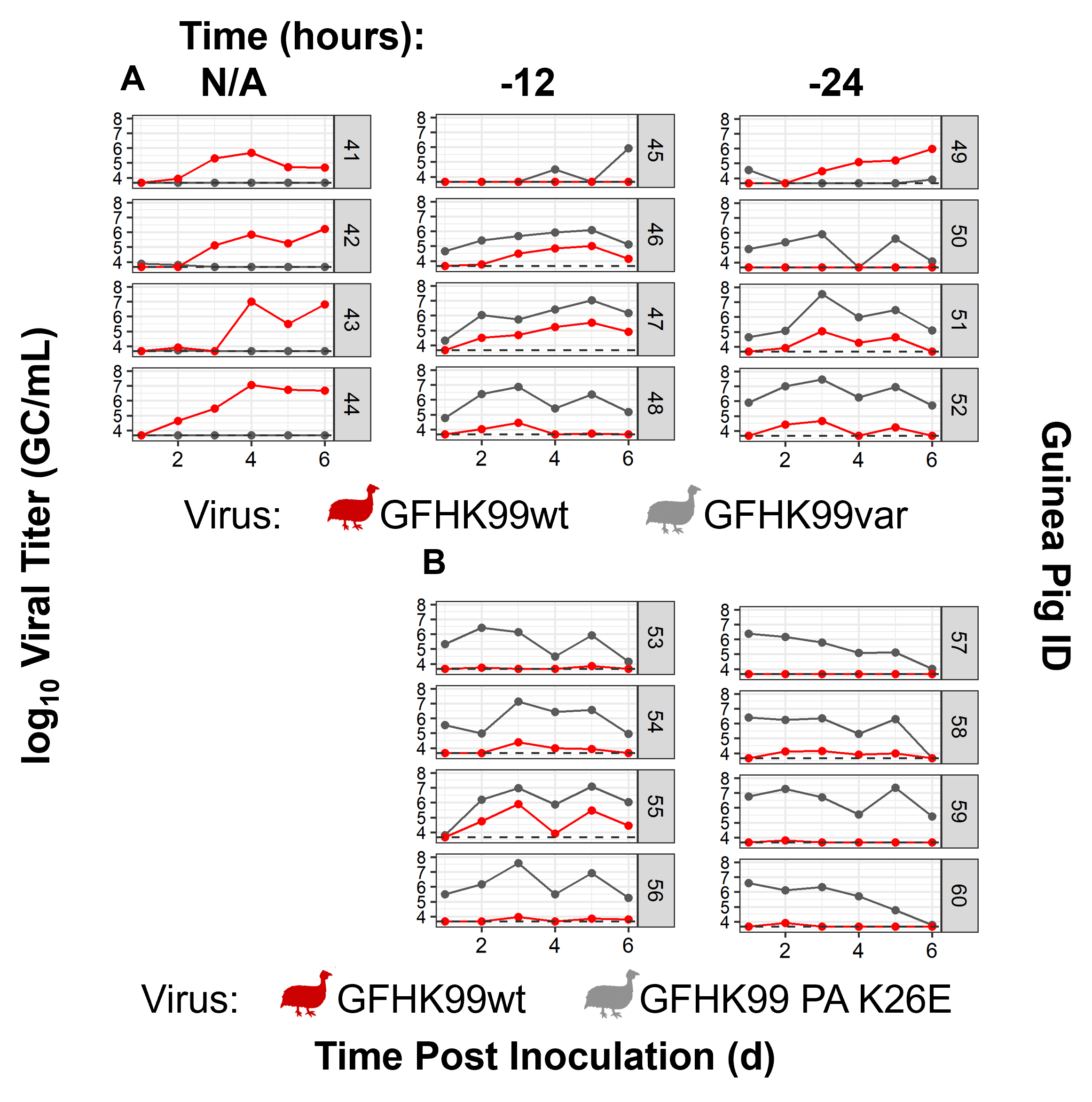

Supplement: S3 Fig — (Related to Fig 4B). Guinea pigs were pre-inoculated with 104 GC of either GFHK99var (A) or GFHK99 PA K26E (B) virus either 12 or 24 h prior to a dose of 104 GC of GFHK99wt virus. The viral titer in nasal washes is plotted and the limit of detection is indicated by the dashed line. Guinea pig ID numbers are shown in grey boxes appended to each facet. (TIF) [file ppat.1010978.s003.tif]

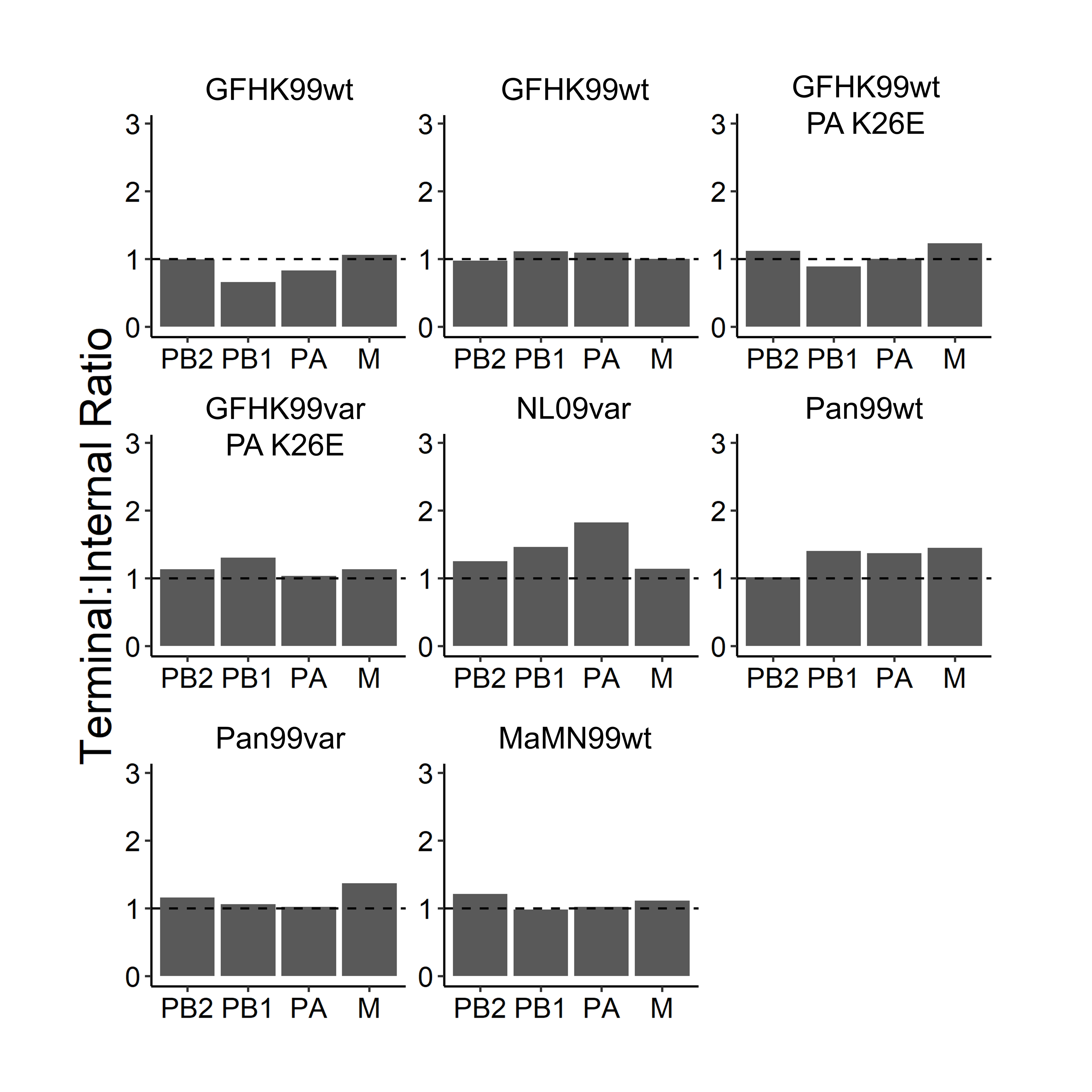

Supplement: S4 Fig — Levels of defective viral genomes (DVGs) were quantified by ddPCR using primers targeting the terminal and internal regions of the PB2, PB1, PA, and M gene segments. Ratios of terminal to internal copies that are <2.0 indicate low DVG content. Virus stocks tested are named above each facet. Two different stocks of GFHK99wt virus were used. (TIF) [file ppat.1010978.s004.tif]
