# Supplementary material for: Influenza A virus coinfection dynamics are shaped by distinct virus-virus interactions within and between cells
Source: PLoS Pathog. 2023 Mar 2;19(3):e1010978. doi: 10.1371/journal.ppat.1010978 (PMC10013887; doi:10.1371/journal.ppat.1010978)
Supplement: S1 Table — (DOCX) [file ppat.1010978.s005.docx]

**Supplementary Table 1. Genotypes of modified viruses.**

|  | **PB2** | **PB1** | **PA** | **HA** | **NP** | **NA** | **M** | **NS** |
| --- | --- | --- | --- | --- | --- | --- | --- | --- |
| **GFHK99var_2_** | A300G,  A303T,  T306C,  G459C,  T461A,  T467T | T282C,  T285C,  A288G,  A420G,  T426C,  C432T | A351G, C354T, C357T, T501G, C504T, C507T | A338G, A344C, A351C, A432G, T435A, C438T | A345G, G351A, A354G, A486T, C489T, A495G | A424G, T430A, G433A, A583G, G586C, G589C | G340A, A343G, A349G, G439A, C442T, A445G | C386T, G389A, A392G, A479G, G482C, A488G |
| **NL09var** | C273T | T288C | C360T | C305T | A351G | G336A | G295A | C341T |
| **Pan99var0** | A345T,  C360T | A540G | G333A, A342G | T308A,  C311A,  C314T,  A464T,  C467G,  T470A | C537T,  T538A,  C539G,  C612G,  G615A | C418G,  T421A,  A424C | G586A | A329T,  A335T,  C341G |
| **GFHK99wt PA K26E** | --- | --- | A100G,  A102G | --- | --- | --- | --- | --- |
| **GFHK99var_2_**  **PA K26E** | A300G,  A303T,  T306C,  G459C,  T461A,  T467T | T282C,  T285C,  A288G,  A420G,  T426C,  C432T | A100G,  A102G, A351G, C354T, C357T, T501G, C504T, C507T | --- | A345G, G351A, A354G, A486T, C489T, A495G | A424G, T430A, G433A, A583G, G586C, G589C | G340A, A343G, A349G, G439A, C442T, A445G | C386T, G389A, A392G, A479G, G482C, A488G |
