# Supplementary material for: Influenza A virus coinfection dynamics are shaped by distinct virus-virus interactions within and between cells
Source: PLoS Pathog. 2023 Mar 2;19(3):e1010978. doi: 10.1371/journal.ppat.1010978 (PMC10013887; doi:10.1371/journal.ppat.1010978)
Supplement: S2 Table — (DOCX) [file ppat.1010978.s006.docx]

**Supplementary Table 2. Primers for the quantification of vRNA by ddPCR.**

| **Universal influenza Reverse Transcription Primers** | |
| --- | --- |
| Univ.F(A)+6 | GCGCGCAGCAAAAGCAGG |
| Univ.F(G)+6 | GCGCGCAGCGAAAGCAGG |
| **GFHK99wt, GFHK99wt PA K26E Virus Primers** | |
| WF10wt NP 336 F | GAAGGAGAGACGGGAAATG |
| WF10wt NP 505 R | GGCTCTTGTTCTCTGGTATG |
| **GFHK99var_2_, GFHK99var_2_ PA K26E Virus Primers** | |
| WF10help NP 388 F | GAAGGAGGGACGGAAAGT |
| WF10help NP 505R | GGGCTCTTGTCCTCTGATAA |
| **NL09var Virus Primers** | |
| NL09 NP 309 F | CCCTAAGAAAACAGGAGGACCC |
| NL09 NP 411 R | TTGGCGCCAAACTCTCCTTA |
| **Pan99wt Virus Primers** | |
| Pan99wt NP 520 F | ATGGATCCCAGAATGTGCTC |
| Pan99wt NP 625 R | TCAGCTCCATTGTC |
| **Pan99var Virus Primers** | |
| Pan99var0 NP 520 F | ATGGATCCCAGAATGTGTAG |
| Pan99var0 NP 625 R | TCAGCTCCATAGTG |
| **MaMN99wt Virus Primers** | |
| MN99 NP 378 F | CGACAAAGAAGAGATCAGAAGGA |
| MN99 NP 457 R | TCATCAAATGGGTGAGACCA |
